# Association of metabolic dysfunction-associated steatotic liver disease with bone health in adults: a systematic review and meta-analysis of observational studies

**DOI:** 10.3389/fendo.2025.1717852

**Published:** 2026-01-12

**Authors:** Xingzhi Li, Wei Luo, Ke Chen, Yong Peng

**Affiliations:** 1 Beijing Anzhen Nanchong Hospital, Capital Medical University & Nanchong Central Hospital, Nanchong, Sichuan, China; 2 The Second Clinical Medical College of North Sichuan Medical College (University), Nanchong, Sichuan, China

**Keywords:** bone mineral density, bone turnover markers, metabolic dysfunction-associated steatotic liver disease, osteoporosis, osteoporotic fractures, systematic review

## Abstract

**Background:**

Several studies have explored the effects of metabolic dysfunction-associated steatotic liver disease (MASLD) on bone mineral density (BMD) and bone turnover markers (BTMs) in adults and the risk of osteoporotic fractures. However, the extent of adverse effects of MASLD on bone health remains uncertain. This systematic review and meta-analysis aimed to investigate the effects of MASLD on bone health in adults.

**Methods:**

We searched PubMed, Medline, EMBASE, Cochrane Library, and Scopus for observational studies published from inception to June 2025 that reported MASLD effects on bone health.

**Results:**

This meta-analysis indicated that MASLD was associated with increased femur BMD (WMD: 0.03 g/cm^2^, 95% CI: 0.02 to 0.03, P<0.001), especially in women (WMD: 0.02 g/cm^2^, 95% CI: 0.01 to 0.04, P = 0.002) and overweight people (WMD: 0.04 g/cm^2^, 95% CI: 0.01 to 0.06, P = 0.002). MASLD was also significantly associated with increased osteoporosis/osteoporotic fractures (OR: 1.23, 95% CI: 1.13-1.34; P<0.001) and decreased BTMs (CTX: WMD: -0.03 ng/mL, 95% CI: -0.05 to -0.02, P<0.001; OC: WMD: -1.86 ng/mL, 95% CI: -2.69 to -1.03, P<0.001; P1NP: WMD: -4.59 ng/mL, 95% CI: -5.64 to -3.54, P<0.001).

**Conclusions:**

MASLD was significantly associated with increased femur BMD compared to non-MASLD patients, especially in women and overweight individuals. Interestingly, increased risk of OP/osteoporotic fractures and decreased BTMs were significantly associated with MASLD. This may suggest that BMD underestimates the risk of OP/osteoporotic fractures in MASLD patients.

## 1 Introduction

Non-alcoholic fatty liver disease (NAFLD) encompasses various liver conditions characterized by fat accumulation without other causes like alcohol or viruses (1). It ranges from simple steatosis to non-alcoholic steatohepatitis (NASH), which involves hepatocyte inflammation, lobular inflammation, and fibrosis, potentially leading to cirrhosis and liver cancer (2). NAFLD imposes a huge health burden that continues to grow globally and in the Asia-Pacific region, affecting about a quarter of the adult population worldwide (3). The diagnosis of NASH requires not only ruling out alcohol, but also histological confirmation of fatty liver disease. The significance of alcohol consumption is often overstated. Recent epidemiological and clinical research indicates the need to distinguish NAFLD from metabolic dysfunction-associated steatotic liver disease (MASLD) (3).

MASLD highlights the significance of metabolic dysfunction and accommodates various etiological factors, regardless of the presence or absence of alcohol consumption and hepatitis infection. MASLD is characterized by hepatic steatosis and plus at least 1 of 5: (1) BMI ≥ 25 kg/m2 (≥ 23 kg/m2 in Asian) or waist circumference > 94 cm in men, > 80 cm in women, or ethnicity adjusted;(2) Fasting serum glucose ≥ 100 mg/dL (≥ 5.6 mmol/L) or 2-hour post-load glucose level ≥ 140 mg/dL (≥ 7.8 mmol/L) or HbA1c ≥ 5.7% or on specific drug treatment;(3) Blood pressure ≥ 130/85 mmHg or specific drug treatment;(4) Plasma triglycerides ≥ 150 mg/dL (≥ 1.70 mmol/L) or specific drug treatment;(5) Plasma HDL cholesterol < 40 mg/dL (< 1.0 mmol/L) for men and < 50 mg/dL (< 1.3 mmol/L) for women or specific drug treatment (4). At the same time, the MASLD framework allows for the exclusion of NAFLD patients who exhibit metabolic dysfunction, thereby enhancing the integration of current knowledge into patient heterogeneity and encouraging the adoption of terminology that more accurately reflects the underlying pathogenesis (5).

Osteoporosis (OP) is a metabolic bone disease characterized by decreased bone mineral density (BMD) and bone quality, as well as impaired bone microstructure, leading to increased bone fragility and fracture risk (6). The pathogenesis of OP is associated with multiple factors, including hormonal changes, calcium and vitamin D deficiency, and increased bone loss due to oxidative stress and osteoclastic bone resorption (7, 8). Low BMD and associated osteoporotic fractures are major health problems that can lead to significant morbidity and mortality (9). Therefore, the identification of therapeutic factors that reduce the risk of OP and improve overall bone health is essential for global public health. Bone turnover markers (BTMs) are biological indicators that reflect the rates of bone formation and resorption, whose levels are indicative of the bone metabolism status. Bone tissue not only provides structural support to the body but also plays a significant role in metabolic diseases (10).

Osteocalcin, a direct indicator of bone formation, regulates energy metabolism. Procollagen Type I n-terminal propeptide (PINP), parathyroid hormone (PTH), and C-terminal cross-linked telopeptide (CTX) are BTMs commonly used in clinical studies and trials (11), and endorsed by both the “International Osteoporosis Foundation” and the “International Federation of Clinical Chemistry”. The detection of serum BTMs offers a clinical method for monitoring bone formation and resorption (12).

However, previous studies on the association between MASLD and BMD have yielded inconsistent results. Some studies indicate that MASLD and its severity are independently correlated with an increased reduction in the values of BMD (13). Lee et al. found that MASLD has different effects depending on the sex of the patient and location of BMD measurement in the patient. BMD at the femoral neck was significantly lower in men with MASLD after adjusting for confounders, while BMD at the lumbar spine was significantly higher in female participants after adjusting for confounders (14). Additionally, other studies have not found any significant association between MASLD and BMD (15). Recent large-scale cross-sectional studies have reported a significant association between MASLD and a self-reported history of osteoporotic fractures in older men, but no such association has been reported in women (16, 17). These findings, similar to those observed in individuals with obesity or type 2 diabetes mellitus, suggest that BMD measurements may underestimate the long-term risk of osteoporotic fractures in patients with MASLD, who frequently present with obesity or diabetes (18).

Therefore, to determine whether this suggestion is correct, we performed a comprehensive systematic review and meta-analysis aimed at exploring the association of MASLD with the prevalence of BMD, and OP and osteoporotic fractures at different skeletal sites in adults, as well as its potential association with BTMs. Additionally, we also conducted subgroup analyses by race, sex, weight and MASLD diagnosing methods. The elucidation of the potential adverse effects of MASLD on bone health could have significant clinical implications for the prevention, diagnosis, and treatment of this prevalent skeletal disorder.

## 2 Methods

### 2.1 Search strategy

A systematic search was conducted on the PubMed, EMBASE, Web of Science, Cochrane Library, and OVID databases from inception to June 2025. The search strategy is described in supplementary materials.

### 2.2 Study selection

Study selection criteria were as follows: (1) Studies on the relationships between MASLD and BMD, BTMs or the prevalence or risk of OP and osteoporotic fractures; (2) The included studies were all observational studies, comprising cohort studies, case–control studies and cross-sectional analyses; (3) The relevant indicators that were available included odds ratio (OR), or hazard ratio (HR), 95% confidence interval (95%CI), the prevalence of OP, the mean and standard deviation (SD) of BMD or BTMs in MASLD patients and non-MASLD patients; (4) MASLD diagnosed using abdominal ultrasound, liver transient elastography, vibration-controlled transient elastography, the International Classification of Diseases (ICD), ultrasonographic Fatty Liver Index (USFLI) and Hepatic Steatosis Index (HSI), liver biopsy, or other markers of MASLD, such as fatty liver index (FLI); (5) Studies whose full text was available; (6) Studies whose age of participants was ≥18 years; (7) The included studies were English literature.

Exclusion criteria were as follows: (1) The study was not performed on human subjects; (2) case reports, commentaries, correspondence, reviews, meta-analysis or systematic evaluations or editorials; (3) the full text of the study was unavailable, lacking complete information and data; (4) participants in the study are taking drugs related to bone metabolism, including patients with various kidney diseases who are taking glucocorticoid, recent fractures, or cancer; study participants had thyroid disease, parathyroid disease, rheumatic immune disease, anorexia and other conditions that affect bone density; (5) other cause-specific liver diseases: autoimmune liver disease, hepatitis, the use of drugs such as methotrexate that specifically cause fatty liver disease, and genetic diseases such as Wilson.

### 2.3 Data extraction and quality assessment

Two authors (WL and XL) independently extracted the usable data from each study into a pre-designed excel table (**Supplementary Table S3**). When there is a difference between two authors (WL and XL) in the independent data extraction process, the dispute is resolved through third-party arbitration (YP).

The methodological quality of the cohort studies was assessed by authors WL and XL using the Newcastle-Ottawa scale (NOS) (**Tables 1**, **S1**).

**Table 1:** Characteristics of participants in included studies.

Exposed/Control							
Author	Country	Age	Subjects	Outcome	Gender	Study design	Adjusted covariates
MASLD and BMD/osteoporosis							
Moon et al. (2012) (34)	Korea	59.5 ± 0.5/58.2 ± 0.7	163/102	Lumbar BMD	Postmenopausal Female	Cross-sectional	age, the presence of metabolic syndrome, ALT, smoking status, alcohol consumption status,
Mian Li et al. (2012)	China	57.2 ± 9.2/60.2 ± 10.4	748/1693	Osteoporotic fracture	Male	Cross-sectional	age, smoking and alcohol consumption habits (yes/no), physical activity (MET-h/wk), eGFR, BMI, waist circumference, serum TG, TC, HDL-c, LDL-c, r HOMA-IR, diabetes status (yes/no), oral steroid use (yes/no), osteoporosis medication (yes/no), rmenopause status (yes/no) and hormone replacement (yes/no)
		58.9 ± 8.7/57.7± 9.9	1604/3752		Female		
Ran Cui et al. (2013)	China	60.63 ± 3.68/58.94 ± 6.66	46/53	Lumbar BMD, rhip BMD, femur neck BMD	Male	Cross-sectional	weight, BMI, waist, HDL, and ALT
		59.89 ± 6.52/58.75 ± 4.95	73/52	Lumbar BMD, hip BMD, femur neck BMD	Female		
Ming Feng Xia (2016)	China	62 (55–70)/63 (56–74)	416/413	Lumbar spine, hip and whole body BMD	Both	Cohort study	liver fat content, age, body weight, alcohol drinking, cigarette smoking, SBP, FBG, triglyceride, cholesterol, HDL-c, uric acid, body fat percentage, and trunk to appendicular fat ratio
S. H. Lee et al. (2016)	Korea	55.511 ± 8.236/57.118 ± 9.078	1288/2018	Lumbar BMD, Femoral neck BMD	Male	Cross-sectional	age, BMI, waist circumference, ALT level, high-sensitivity C-reactive protein level, smoking status, alcohol consumption status, physical activity, diastolic blood pressure, fasting plasma glucose, triglyceride level, high-density lipoprotein level, eGFR
		62.933 ± 6.211/62.764 ± 6.421	1217/2112	Lumbar BMD, femoral neck BMD	Female		
Yanmao Wang et al. (2018)	China	72.97 ± 5.63/74.71 ± 5.93	226/724	Osteoporotic fracture	Male	Cross-sectional	age, r smoking status, alcohol status, r physical activity. diabetes, hypertension, cardiovascular events, family history of fracture, waistline, BMI, loss weight >7%, r serum TG, TC, HDL-c, LDL-c
		72.10 ± 5.42/73.27 ± 6.24	388/1321		Female		
Dong-Yun Lee et al. (2018)	Korea	54.9 ± 2.5/54.4 ± 2.5	605/3134	Lumbar BMD, femoral neck BMD	Postmenopausal female	Cross-sectional	Age, age at menarche, years since menopause, body mass index, alanine aminotransferase, alkaline phosphatase, HbA1C, homeostasis model assessment of insulin resistance, estimated glomerular filtration rate, uric acid, potassium, estradiol, osteocalcin, and C-telopeptide
Da-Zhi Chen et al. (2018)	China	61.21 ± 13.81	64/641	Osteoporosis	Postmenopausal Female	Cross-sectional	Age, TC, ALP, MetS
Hon-Jhe Chen et al. (2018)	China	44.94 (35.60–54.94)/44.94 (35.60–54.92)	4318/17272	Osteoporosis	Male and Female	Cohort study	Age, sex, comorbidities, degree of urbanization, income
Umehara et al. (2018)	America	55.6/54	1690/4399	Femoral neck BMD	Male and Female	Cross-sectional	Age, BMI, Ethnicity, sex, menopause
Sung et al. (2020)	Korea	49.6 ± 9.3/49.0 ± 10.2	434/872	Osteoporosis, Femoral neck BMD	Male	Cohort study	age, sex, BMI, hypertension, diabetes, history of dyslipidemia, HOMA-IR, hsCRP, current smoking status, alcohol intake, vigorous physical activity
	Korea	47.6 ± 8.1/42.6 ± 7.5	446/3084		Female	Cohort study	
Zhe Shen et al. (2020)	China	51.2 ± 9.9	473/1247	Osteoporosis	Male and Female	Cohort study	age, gender, BMI, Ca, smoking statue (never, past and current smokers)
Ciardullo et al. (2021)	Italy	62.9 ± 0.7/63.2 ± 0.7	488/437	Osteopenia/ Osteoporosis	Male	Cross-sectional	BMI, body mass index; CAP, controlled attenuation parameter
	Italy	63.1 ± 0.7/63.3 ± 0.7	391/468		Female		
Yoon et al. (2021)	Korea	60.1 ± 7.1/58.0 ± 7.3	888/1735	Total hip BMD	Male and Female	Cohort study	age, (sex), body mass index, smoking, waist circumference, hypertension, diabetes, triglyceride, HDL cholesterol and LDL cholesterol
Tianyu Zhai et al. (2021)	America	58.37 ± 0.73/56.62 ± 0.49	381/813	Femoral neck BMD, Lumbar spine BMD, Osteoporotic fracture	Male and Female	Cross-sectional	ex, age, race, BMI, waist circumference smoking, educational, marital, economic status, nutritional status, 25(OH)D, milk intake, r hypertension, diabetes, HDL-C, TG, TC, LDL-C and physical activity
Ruijie Xie et al. (2022)	China	40.665 ± 11.886/36.835 ± 11.891	770/1210	Lumbar BMD	Male and Female	Cross-sectional	Age, gender, race, body mass index, poverty to income ratio, education, smoking behavior, Moderate activities, Diabetes status, Waist circumference, HbA1c (%), Total cholesterol, Triglyceride, LDL- cholesterol, HDL- cholesterol, ALT, ALP, GGT, AST, Serum creatinine, Serum iron, Lumbar bone mineral density, CAP and LSM
Loosen et al. (2022)	Germany	59.5 ± 13.9/59.5 ± 13.9	50689/50689	Osteoporosis and Osteoporotic fracture	Male and Female	Cohort study	Age, sex, obesity
Yi-Jun Du et al. (2022)	China	63.17 ± 9.37/63.29 ± 8.93	198/236	Femoral neck BMD, Lumbar spine BMD, total Hip BMD	Female	Cross-sectional	/
Hejun Li et al. (2022)	China	62.89 ± 0.47/63.24 ± 0.47	846/1185	Total femur BMD, Femur neck BMD, Trochanter BMD, Intertrochanter BMD	Male and Female	Cross-sectional	age, sex, race/ethnicity, education, marital status, dring habit, uric acid, Phosphorus, alkaline phosphatase, creatinine, alanine aminotransferase, copd, cancer, maternal fracture history, family history of osteoporosis,
		62.89 ± 0.47/63.24 ± 0.47	846/1185	Osteoporosis			age, sex, race/ethnicity, education, marital status, dring habit, uric acid, Phosphorus, alkaline phosphatase, creatinine, alanine aminotransferase, copd, cancer, family history of osteoporosis
Wester et al. (2022)	Sweden	55(41-65)/55(40-65)	10678/99176	Osteoporotic fracture	Male and Female	Cohort study	patient-control strata with no further adjustments, birth, rheumatic disease, chronic obstructive pulmonary disease, dementia, cancer, chronic kidney disease, cardiovascular disease, diabetes, and obesity
Jinmin Liu et al. (2023)	China	63.60 ± 8.64/64.56 ± 9.08	381/436	Total femur BMD, Femur neck BMD, Trochanter BMD, Intertrochanter BMD	Male and Female	Cross-sectional	age, sex, race/ethnicity, ALT, AST, GGT, cholesterol, calcium, ALB, P, glycohemoglobin, platelet, vitamin D intake, calcium intake, smoking status, family history of osteoporosis, cancer, chronic obstructive pulmonary disease, heart failure, and use of glucocorticoid.
Hansen et al. (2023)	Denmark	46.4± 11.8/40.5± 11.0	37/32	Lumbar Spine BMD,Total Hip BMD	Male and Female	Cross-sectional	Age, sex. BMI, diabetes
Wei Zhang et al. (2023)	China	54.9 ± 13.0/58.8 ± 10.6	144/105	Osteoporosis	Male and Female	Cross-sectional	age, sex, ever smoking and drinking, systolic blood pressure, diastolic blood pressure, diabetes duration, HbA1c, diabetes medical treatment, total cholesterol, triglycerides, HDL-C, LDL-C, and BMI.
Guangheng Zhang et al. (2023)	China	70.6 ± 4.7/71.6 ± 6.0	332/384	Osteoporosis	Male and Female	Cross-sectional	age, sex, systolic blood pressure, diastolic blood pressure, overweight rate, history of hypertension, history of diabetes, history of smoking, history of alcohol consumption, fasting glucose, hemoglobin, urea, creatinine, triglycerides, total cholesterol, high-density lipoprotein cholesterol, low-density lipoprotein cholesterol, waist circumference, total protein, albumin, and globulin
Goh Eun Chung et al. (2023)	Korea	59.5 ± 7.6/60.4 ± 8.5	438525/1988130	Osteoporotic fracture	Male and Female	Cohort study	age, sex, body mass index, income, smoking, alcohol consumption, regular exercise, hypertension, diabetes, and dyslipidemia
Hsiao-Yun Yeh et al. (2024)	China	63.48 ± 9.33/66.59 ± 10.72	215/226	Osteoporosis	Female	Cohort study	Age, BMI, TG, HDL-C, uric acid, and ALT
		63.86 ± 10.16/68.89 ± 11.58	238/262		Male		
Binjing Pan et al. (2024)	China	63.42 ± 8.37/61.67 ± 7.40	277/423	Total hip BMD, L4 BMD, L1–4 BMD	Male aged ≥ 50 years	Cross-sectional	age, BMI, and diabetes duration; AST, ALT, TBIL, TC, TG, LDL-C, 25- (OH) VitD, FT3, HOMA-IR
		64.22 ± 8.50/63.84 ± 8.75	183/26		Postmenopausal female		
Alpesh Goyal et al. (2024)	India	32.2 ± 5.1/31.5 ± 4.9	170/124	Osteoporosis, Lumbar BMD, total BMD, Femoral neck BMD	Female	Cross-sectional	age, postpartum interval, exclusive breastfeeding ≥6 months, history of GDM, current prediabetes, serum 25(OH)D level, dietary calcium intake, BMI and physical activity
MASLD and BTMs							
Ran Cui et al. (2013)	China	60.63 ± 3.68/58.94 ± 6.66	46/53	CTX, PTH, OC	Male	Cross-sectional	/
		59.89 ± 6.52/58.75 ± 4.95	73/52	CTX, PTH, OC	Female		
Jun-Jie Liu et al. (2013)	China	40.52 ± 10.18/36.69 ± 11.37	364/1319	OC	Male	Cross-sectional	/
Jianxin Dou et al. (2013)	China	53.32 ± 8.3/54.3 ± 8.8	449/1109	OC	Male	Cross-sectional	/
Yu-qi Luo et al. (2015)	China	56.0 ± 5.0/56.2 ± 4.4	130/603	OC	Female	Cross-sectional	/
Ming Feng Xia (2016)	China	62 (55–70)/63 (56–74)	416/413	OC, CTX	Both	Cohort study	/
Hae Jin Yanga et al. (2016)	Korea	45 ± 6/45 ± 7	249/610	OC	Male	Cross-sectional	/
Dong-Yun Lee et al. (2018)	Korea	54.9 ± 2.5/54.4 ± 2.5	605/3134	OC, CTX	Postmenopausal female	Cross-sectional	/
H Deng et al. (2018)	China	50.52 ± 4.87/50.35 ± 5.18	232/308	OC, CTX, P1NP	Male	Cross-sectional	/
Da Fang et al. (2022)	China	53.0 (48.0, 57.0)/52.0 (47.0, 57.0)	75/295	OC, CTX, P1NP, PTH	Male and Female	Cross-sectional	/
Sichao Wang et al. (2022)	China	57 (48,66)/64 (58,70)	129/57	OC, CTX, P1NP	Male and Female	Cross-sectional	/
J.Fu et al. (2022)	China	63.40 ± 10.03/66.71 ± 9.08	552/474	OC, CTX, P1NP	Male and Female	Cross-sectional	/
Zhiyan Yu et al. (2022)	China	26.58 ± 3.23/23.89 ± 3.15	388/266	OC, CTX, P1NP, PTH	Male	Cross-sectional	/
		26.10 ± 3.76/23.88 ± 3.89	372/217		Female		/
Chongyang Chen et al. (2024)	China	58.64 ± 9.88/62.30 ± 9.46	306/436	OC, CTX, P1NP	Male and Female	Cross-sectional	/
Alpesh Goyal et al. (2024)	India	32.2 ± 5.1/31.5 ± 4.9	170/124	OC, CTX, P1NP	Female	Cross-sectional	/

BMI, Body mass index, AST, Acid aminotransaminase, ALT, Alanine aminotransaminase, TBIL, Total ilirubin, TG, triglycerides, TC, Total cholesterol, HDL-C, High density lipoprotein cholesterol, LDH, Lactic ehydrogenase, Ca, Calcium, P, Phosphorus, 25- (OH) VitD:25-hydroxy vitamin D, FT3, free triiodothyronine, HbA1c, Glycosylated hemoglobin, HOMA-IR, Homeostasis model assessment of insulin resistance, BTMs, Bone Turnover Markers, OC, Osteocalcin; PINP, Type I procollagen amino-terminal peptide; CTX, β-Type I collagen carboxy-terminal peptide, PTH, parathyroid hormone, CAP, Controlled attenuation parameter, LSM, Liver stiffness measurement.

34th reference.

The quality of cross-sectional studies was assessed using 11 checklists recommended by the Agency for Health Care Research and Quality (AHRQ) (**Supplementary Table S2**) (19). According to the overall quality of cross-sectional studies, the ratings were moderate to good (4-11) or poor (3- 0).

### 2.4 Statistical analysis

For each study, the OR and HR values, along with their95%CI, were transformed into their natural logarithms. These logarithmic transformations of the OR and HR values, and their corresponding 95%CIs were subsequently used to assess the association between MASLD and the prevalence of OP and osteoporotic fractures. The mean ± SD values of the BMD and BTMs were calculated to estimate the combined effect using the weighted mean difference (WMD).

Heterogeneity was assessed by the I2 statistic and Cochran’s Q statistic, with I2 > 50% and p<0.01 indicating significant heterogeneity.

Interquartile ranges (IQRs) are used to show where the spread or dispersion of the middle 50% of the data points. For large samples with a normal distribution, the IQR is about 1.35 standard deviations (SDs) wide. However, when distributions are skewed, estimating the SD from IQRs is not feasible. The use of IQRs instead of SD often suggests a skewed distribution. Wan and colleagues developed a size-dependent formula to approximate SD from IQRs (20).

A sensitivity analysis was performed to evaluate the impact of each study by systematically excluding individual studies and assessing their influence on the overall cumulative risk estimates. Egger’s and Begg’s linear regression models were used to assess publication bias. The two-tailed P value was less than 0.05, and the difference was statistically significant. Subgroup analyses were performed based on differences in the BMD values of the experimental group population, MASLD diagnosis method, sex, geographic region and overweight. All collected data were statistically analyzed using the STATA 15.0 software (StataCorp, College Station, TX, USA).

## 3 Results

### 3.1 Study selection, characteristics, and quality assessment

The characteristics of the included observational studies are shown in **Table 1**, and detailed risk assessment information is provided in **Supplementary Tables S1** and **S2**. All studies were assessed to be of moderate to good quality. The correlation data between MASLD and BMD, BTMs, and OP and osteoporotic fractures in each of the included studies are shown in **Supplementary Table S3**. In the current systematic review and meta-analysis, the prevalence of OP and osteoporotic fractures was assessed in 16 studies including 2,684,403 participants (13, 16, 17, 21–33). The effect of MASLD on BMD values was assessed in 16 studies including 28839 participants (14, 24, 25, 27, 32, 34–44), and the effect of MASLD on BTMs was assessed in 14 studies including 14,026 participants (32, 35–37, 45–53). The studies were from 8 different countries (China, Korea, USA, Italy, Germany, Sweden, Denmark and India). The steps of the literature search program process are summarized in **Supplementary Figure S1**.

### 3.2 MASLD and BMD values

**Figure 1:**
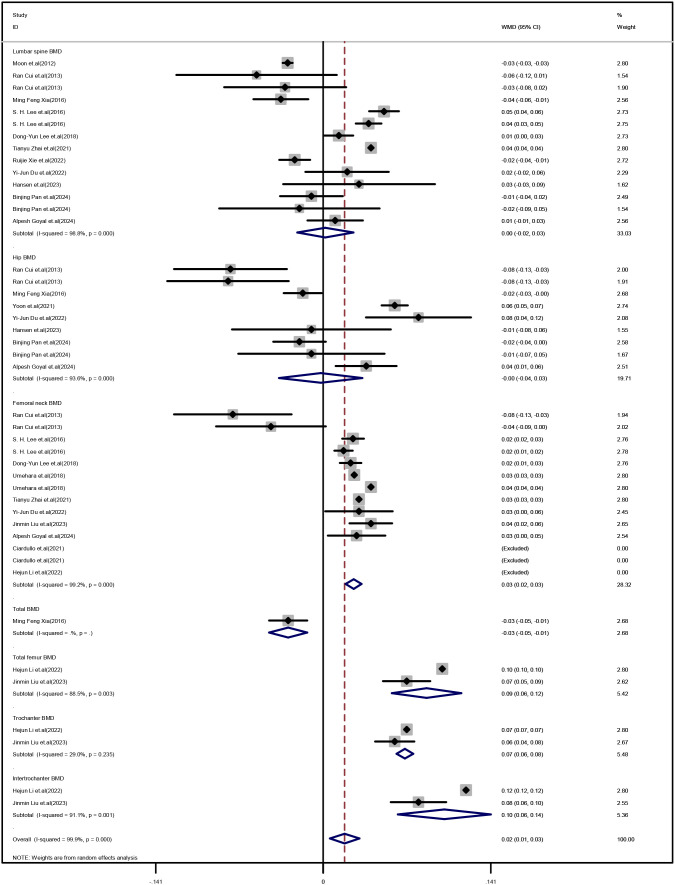
Forest plot of comparison of BMD values at different skeletal sites between the MASLD and control groups.

Our meta-analysis showed no significant difference in lumbar spine BMD (WMD: 0.002 g/cm2, 95%CI: -0.02 to 0.03, Z = 0.14, P = 0.885; I2 = 98.8%) and hip BMD (WMD:-0.002 g/cm2, 95%CI: -0.04 to 0.03, Z = 0.12, P = 0.906; I2 = 93.6%) between MASLD patients and non-MASLD patients. Remarkably, femoral BMD was significantly higher in MASLD patients than in non-MASLD patients (femoral neck BMD: WMD: 0.03 g/cm2, 95%CI: 0.02 to 0.03, Z = 7.54, P<0.001, I2 = 99.2%; total femur BMD: WMD: 0.09 g/cm2, 95%CI: 0.06 to 0.12, Z = 5.82, P<0.001, I2 = 88.5%; trochanter BMD: WMD: 0.07 g/cm2, 95%CI: 0.06 to 0.08, Z = 19.36, P<0.001, I2 = 29%; intertrochanter BMD: WMD: 0.1 g/cm2, 95%CI: 0.06 to 0.14, Z = 5.11, P<0.001, I2 = 91.1%) [**Figure 1]**.

Although our study found that among overweight subjects, MASLD patients had higher overall total BMD values than non-MASLD patients (WMD: 0.02 g/cm2, 95%CI: 0.001 to 0.03, Z = 2.07, P = 0.039, I2 = 99.9%) [**Supplementary Figure S4(a)**], after stratifying BMD values by site, we found that MASLD patients had higher femur BMD values (WMD: 0.04 g/cm2, 95%CI: 0.01 to 0.06, Z = 3.08, P = 0.002, I2 = 100%) [**Supplementary Figure S4(b)**], compared with non-MASLD patients. Lumbar spine BMD values and hip BMD values did not differ between overweight or normal weight MASLD patients and non-MASLD patients[**Supplementary Figure S4(c)**, **S4(d)**]. Additionally, we also found higher femoral BMD values in females with MASLD when we stratified by sex (WMD: 0.02 g/cm2, 95%CI: 0.01 to 0.04, Z = 3.1, P = 0.002, I2 = 91.9%) [**Supplementary Figure S2(b)**]. However, total BMD, lumbar BMD and hip BMD values did not differ significantly between MASLD patients and non-MASLD patients [**Supplementary Figures S2(a, c, d)**].

Furthermore, total BMD values were higher in MASLD patients than in non-MASLD patients in both Asian (WMD: 0.02g/cm2, 95%CI: 0.005 to 0.029, Z = 2.71, P = 0.007; I2 = 99.7%) [**Supplementary Figure S3(a)**] and non-Asian populations (WMD: 0.03, 95%CI: 0.03 to 0.04, Z = 7.91, P<0.001; I2 = 99.6%) [**Supplementary Figure S3(a)**], especially in femur BMD values (Asian: WMD: 0.05 g/cm2, 95%CI: 0.03 to 0.06, Z = 5.91, P<0.001; I2 = 99.8%; non-Asian: WMD: 0.03 g/cm2, 95%CI: 0.02 to 0.04, Z = 6.38, P<0.001; I2 = 99.8%) [**Supplementary Figure S3(b)**]. Moreover, we found higher lumbar BMD values in the MASLD population than in non-MASLD patients in the non-Asian population (WMD: 0.04 g/cm2, 95%CI: 0.04 to 0.04, Z = 46.18, P<0.001; I2 = 0.00%) [**Supplementary Figure S3(d)**]. Hip BMD values were not significantly different between MASLD and non-MASLD patients [**Supplementary Figure S3(c)**]. Differences in the association between MASLD and BMD values were evident in the stratification of the different diagnostic modalities of MASLD (Ultrasonography (USG)(n=8): WMD: 0.01 g/cm2, 95%CI: -0.001 to 0.01, Z = 1.66, P = 0.098, I2 = 99.1%; vibration-controlled transient elastography (VCTE)(n=5): WMD: 0.07 g/cm2, 95%CI: 0.05 to 0.08, Z = 6.87, P<0.001, I2 = 99.9%; USFLI and HIS (n=1): WMD: 0.03 g/cm2, 95%CI: 0.03 to 0.04, Z = 7.0, P<0.001, I2 = 98.9%; Clinical Diagnostic (n=1): WMD: 0.04 g/cm2, 95%CI: 0.01 to 0.07, Z = 2.5, P = 0.013, I2 = 58.4%; Liver biopsy and histological analyses (n=1): WMD: 0.01 g/cm2, 95%CI: -0.04 to 0.06, Z = 0.46, P = 0.643, I2 = 0.0%; USG and FibroScan (n=1): WMD: 0.02 g/cm2, 95%CI: 0.01 to 0.04, Z = 3.1, P = 0.002, I2 = 15.1%) [**Supplementary Figure S5**].

Sensitivity analysis was performed by eliminating one study at a time, and the results showed that WMD 0.018 g/cm^2^ (0.006 to 0.03) had no significant fluctuation [**Supplementary Figure S6**]. We determined that the P values for Egger’s and Begg’s linear regression were 0.786 and 0.92, respectively [**Supplementary Table S4**], indicating that there was no significant publication bias.

### 3.3 MASLD and OP

**Figure 2:**
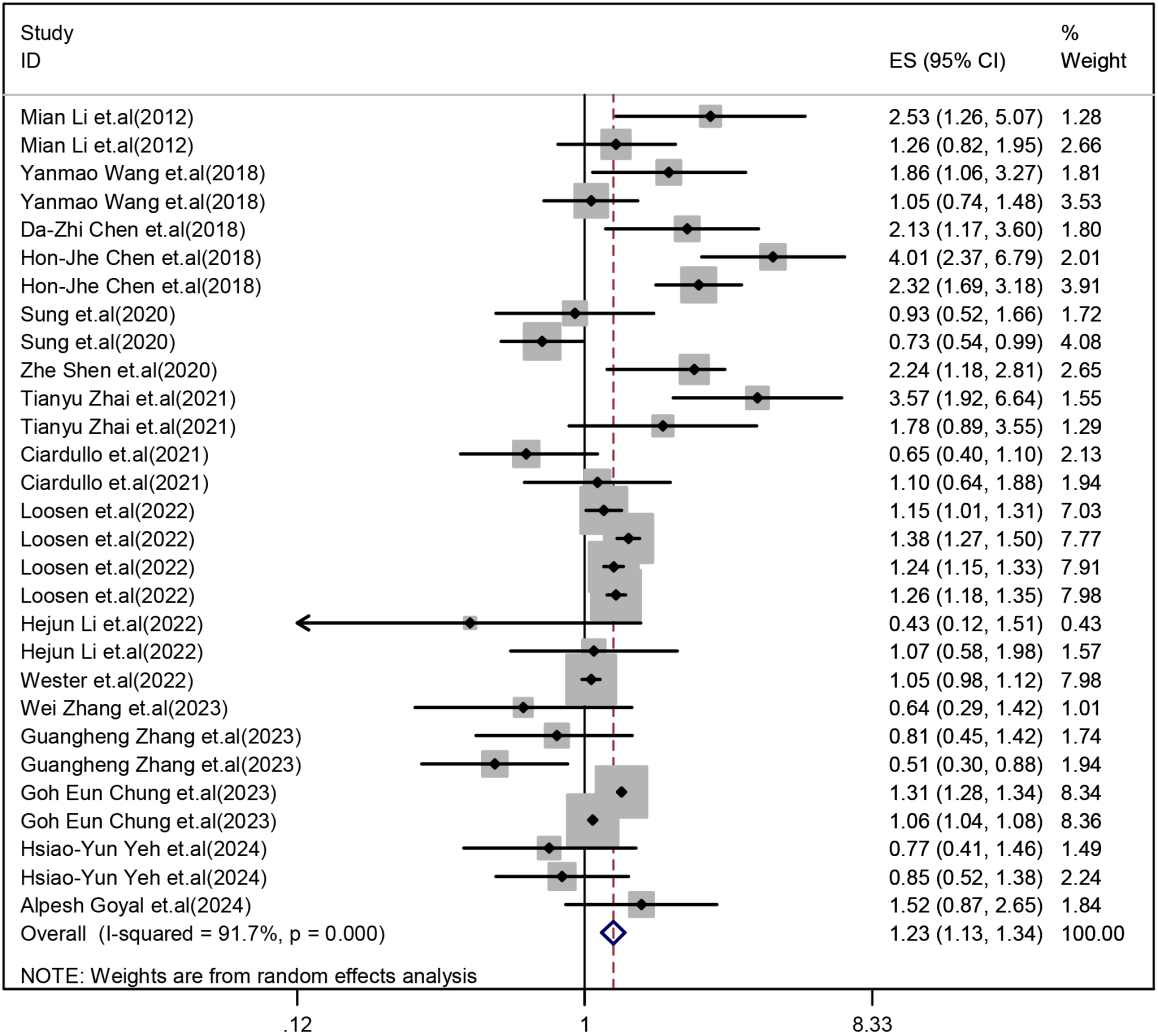
Comparison of the prevalence of OP and osteoporotic fractures between the MASLD and non-MASLD groups.

Our meta-analysis showed that the MASLD group had a higher prevalence of OP and osteoporotic fractures than the non-MASLD group (OR:1.23, 95%CI:1.13 to 1.34, Z = 4.83, P<0.001, I^2^ = 91.7%) [**Figure 2]**.

Subgroup analyses were performed based on sex, weight, diagnostic modalities of MASLD and region.

The results also revealed that the prevalence of OP and osteoporotic fractures was higher in both male patients (OR: 1.25, 95%CI: 1.09 to 1.42, Z = 3.23, P = 0.001, I2 = 77.6%) and female patients (OR:1.18, 95%CI:1.03 to 1.35, Z = 2.44, P = 0.015, I2 = 88.2%) of the MASLD groups than that in the subjects of the non-MASLD group [**Supplementary Figure S7**]. Patients with MASLD had a higher prevalence of OP and osteoporotic fractures than non-MASLD patients in both Asian (OR:1.26, 95%CI: 1.11 to 1.43, Z = 3.58, P<0.001, I2 = 94%) and non-Asian (OR:1.21, 95%CI:1.09 to 1.35, Z = 3.49, P<0.001, I2 = 80.3%) populations [**Supplementary Figure S8**]. Notably, the prevalence of OP and osteoporotic fractures was higher in the MASLD group than in the non-MASLD group in both overweight (OR:1.16, 95%CI:1.02 to 1.33, Z = 2.21, P = 0.027, I2 = 93.7%) and normal weight (OR: 2.20, 95%CI: 1.56 to 3.1, Z = 4.49, P<0.001, I2 = 0.00%) subgroups [**Supplementary Figure S9**]. Differences in the prevalence of OP and osteoporotic fractures in the MASLD group were evident in the stratification of the different diagnostic modalities of MASLD (USG (n=7): OR:1.16, 95%CI: 0.86 to 1.58, Z = 0.96, P = 0.336, I2 = 76.6%; ICD (n=3): OR:1.36, 95%CI:1.19 to 1.54, Z = 4.61, P<0.001, I2 = 90.8%; USFLI and HIS (n=1): OR:2.57, 95%CI:1.3 to 5.07, Z = 2.71, P = 0.007, I2 = 53.6%; VCTE (n=2): OR:0.85, 95%CI: 0.6 to 1.2, Z = 0.93, P = 0.354, I2 = 17%; FLI (n=1): OR: 1.18, 95%CI: 0.96 to 1.45, Z = 1.55, P = 0.121, I2 = 99.5%; liver imaging (n=1): OR:0.82, 95%CI: 0.55 to 1.2, Z = 1.02, P = 0.308, I2 = 0.00%) [**Supplementary Figure S10**].

Sensitivity analysis was performed by eliminating one study at a time and the results showed that OR 1.23 (1.1 to 1.38) had no significant fluctuation (**Supplementary Figure S11**). We also determined that the P values for Egger’s and Begg’s linear regression were 0.465 and 0.639, respectively (**Supplementary Table S5**), indicating that there was no significant publication bias.

### 3.4 MASLD and BTMs

**Figure 3:**
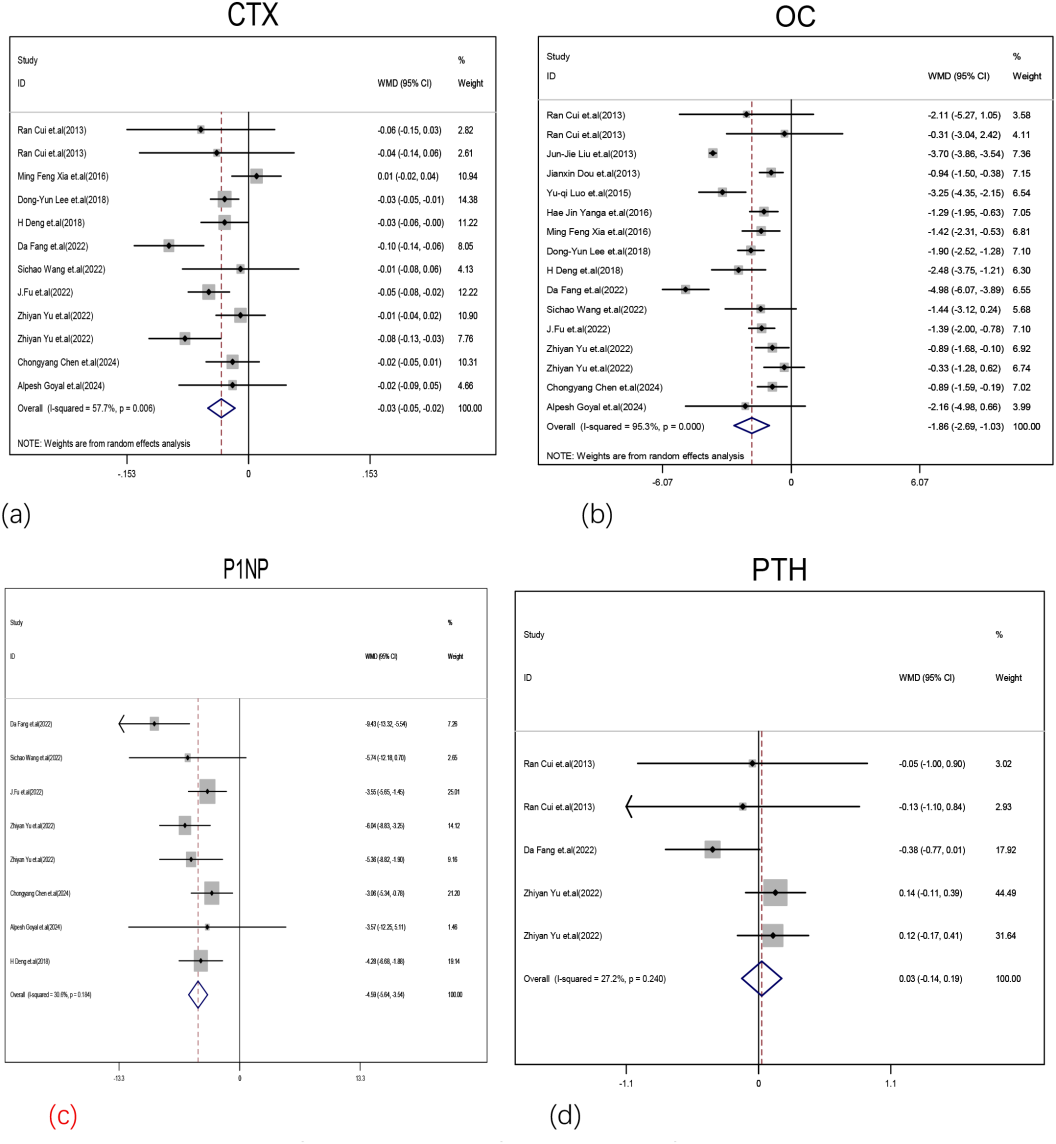
Forest plot of comparison of the levels of BTMs between the MASLD and control groups. **(a)** Forest plot of CTX levels and MASLD. **(b)** Forest plot of OC levels and MASLD. **(c)** Forest plot of P1NP levels and MASLD. **(d)** Forest plot of PTH levels and MASLD.

Our meta-analysis found that the CTX (WMD: -0.03 ng/mL, 95%CI: -0.05 to -0.02, Z = 3.93, P<0.001, I2 = 52.7%), OC (WMD: -1.86 ng/mL, 95%CI: -2.69 to -1.03, Z = 4.37, P<0.001, I2 = 52.7%), and PINP (WMD: -4.59 ng/mL, 95%CI: -5.64 to -3.54, Z = 8.57, P<0.001, I2 = 30.6%) levels were significantly lower in MASLD patients than in controls [**Figures 3a-c**]. There was no significant difference in PTH levels (WMD: -0.03 pmol/L, 95%CI: -0.14 to 0.19, Z = 0.32, P = 0.75, I2 = 27.2%) between the MASLD and control groups [**Figure 3d**].

Sensitivity analysis was performed by eliminating one study at a time, and the results revealed that CTX (WMD: -0.03 ng/mL (-0.06 to -0.01)), OC (WMD: -1.85 ng/mL (-2.8 to -0.78)), PINP (WMD: -4.59 ng/mL (-6.18 to -3.12)) and PTH (WMD: -0.002 pmol/L (-0.4 to 0.3)) levels showed no significant fluctuation [**Supplementary Figures S12(a-d)**]. We also determined the P values for Egger’s and Begg’s linear regression for CTX, P1NP and PTH levels [**Supplementary Tables S6(a, c, d)**], which indicated that no significant publication bias was found. Although Egger’s regression analysis on OC levels yielded a P-value of 0.006 [**Supplementary Table S6(b)**], we promptly performed trim-and-fill analyses and determined that publication bias did not influence our findings, as the P-values of the combined effect sizes remained consistent before and after the analyses. Additionally, we observed no significant differences in heterogeneity or pooled results pre- and post-trim-and-fill analyses, indicating the robustness of our results [**Supplementary Figure S13**].

## 4 Discussion

The findings of our Meta-analysis were as follows: (a) lumbar BMD and hip BMD values were not significantly different between MASLD patients and non-MASLD patients, and remarkably, femoral BMD was significantly higher in MASLD patients than in non-MASLD patients, especially in female and overweight populations; (b) the prevalence of OP and osteoporotic fractures was higher in the MASLD group than in the non-MASLD group, which is consistent with subgroup analyses by country, sex, and weight; (c) CTX, OC and PINP levels were significantly lower in MASLD patients than in controls.

Two meta-analyses reported no significant association between MASLD and BMD values (54, 55). The meta-analysis by Su et al. found a significant adverse effect of MASLD on BMD. However, their meta-analysis included only adjusted effect sizes, which may explain the discrepancies between their findings and those of previous studi. In fact, if only the adjusted results were included, the meta-analysis may lead to sample size reduction and bias in the results (56).

Patients with MASLD typically have higher body weight and BMI, which may increase bone load due to metabolic abnormalities and are considered protective factors against BMD decline (14, 34, 35, 47). Indeed, excess body fat mass may exert greater static mechanical stress on bones, promoting cortical bone formation in obese individuals (57). In addition, higher body fat may increase the secretion of various hormones, including estrogen (58), insulin (59), and leptin (60), which may halt bone loss (61). Therefore, the adverse effects of MASLD on BMD may be offset by the positive effects of high BMI (62). Our subgroup analyses revealed that overweight individuals with MASLD had higher femur BMD compared to those without MASLD, supporting the notion that higher BMI may protect BMD. Additionally, lumbar BMD values were significantly higher in non-Asian MASLD patients than in non-MASLD patients, possibly due to the usually higher BMI in European populations compared to Asian populations.

In our previous subgroup analyses focusing on female participants, we observed that the positive correlation between MASLD and femoral BMD was mostly evident in women aged over 50 years. This finding may be attributed to hormonal factors, particularly the role of estrone. Age-related differences could also play a significant role, as aromatase enzymes present in adipose tissue convert androstenedione to estrone or estradiol, which are critical components of estrogen in postmenopausal women (63). Aromatase activity in adipose stromal cells increases with age, being higher in postmenopausal than premenopausal women (64), and may protect against decrease in BMD.

While our study found that MASLD did not increase the risk of low BMD, it did not demonstrate a beneficial effect on bone metabolism. Furthermore, this study solely assessed the relationship between MASLD and BMD values, without considering the potential effects of MASLD on bone quality. Therefore, we cannot ascertain whether MASLD influences bone metabolism through the impairment of bone quality. We thus conducted a further meta-analysis of the effect of MASLD on OP as well as the levels of BTMs, and found that the levels of BTMs (OC, PINP, β-CTX) were significantly lower in MASLD patients than in non-MASLD patients. Furthermore, the prevalence of OP and osteoporotic fractures was significantly higher in MASLD patients than in non-MASLD patients, and the results held true in subgroup analyses by sex, weight, and country.

Dynamic signaling between osteoblasts and osteoclasts drives the balance between bone formation and resorption, thereby maintaining the balance between mineralization and bone structural integrity. An imbalance between bone resorption and bone formation results in bone loss (65). BTMs are released during the processes of bone formation and resorption, and the OC secreted by osteoblasts during bone formation is involved in matrix mineralization and osteoblast differentiation and thus maintains bone formation and normal mineralization. PINP is secreted as a degradation product generated during the formation of type I collagen by osteoblasts, as well as osteoclasts, and β-CTX is a peptide fragment during the degradation of mature type I collagen secreted by osteoclasts, thus PINP and β-CTX are markers of bone formation and resorption (66). Compared to BMD, levels of BTMs can reflect the state of bone metabolism timely and accurately (66). Bone Formation markers have been shown to be important reference markers for the diagnosis and treatment of osteoporosis and osteoporotic fractures (67). Changes in PINP levels during early treatment correlate with changes in lumbar BMD values at 18 months of treatment (68). Additionally, studies have shown that OC activity is greatly increased after treatment of OP (69). More importantly, many studies have shown that the levels of BTMs are associated with low BMD and OP risk in MASLD patients (37, 47, 70, 71).

Metabolic syndrome may play a key role in MASLD and bone metabolism (72). MASLD represents a hepatic manifestation of metabolic syndrome, contributing to diverse glycolipid metabolic disorders and aberrant bone metabolism. A cross-sectional study showed that participants with dysglycemia had a significantly lower TBS. In addition, participants with dysglycemia had significantly lower serum osteocalcin levels (73). Multiple meta-analyses have shown that markers of bone formation and bone resorption are reduced in patients with diabetes. This suggests that diabetes is a state of low bone turnover, which in turn may lead to weaker bones (74, 75). In addition, it may be linked to an increased risk of osteoporotic fractures (76). Some studies have reported that MASLD is associated with an increased risk of self-reported osteoporotic fractures, but not with low BMD values (54, 77). These findings imply that MASLD may exacerbate insulin resistance and trigger the release of proinflammatory cytokines and bone regulatory factors, thereby promoting OP.

In chronic inflammation, proinflammatory cytokines, such as IL-6, IL-1, and TNF, activate osteoclasts, leading to bone resorption. IL-6 and IL-1 directly boost osteoclast function and generation, and indirectly by increasing RANKL production in osteoblasts (78). Increased IL-6 levels during liver injury can promote liver regeneration and may influence bone remodeling in different liver diseases (78).

Reduced IGF-1 levels play a key role in the development of MASLD and OP. In liver fibrosis patients, decreased liver function or liver dysfunction may contribute to OP. Portal shunting and insulin resistance can significantly lower IGF-1 levels, decrease osteoblast activity, and increase bone resorption, thereby disrupting bone balance (79). As IGF-1 and growth hormone levels decrease with age, bone metabolic disorders and OP become more likely, increasing fracture risk (80). IGF-1 may contribute to bone remodeling by activating mTOR-induced osteoblast differentiation, cell migration, and chemotaxis, potentially recruiting mesenchymal stem cells (81). This explains the increased risk of OP and osteoporotic fractures observed in our study, even though MASLD did not increase the risk of low BMD.

Subgroup analysis shows that the risk of OP/osteoporotic fractures in normal-weight MASLD patients (OR: 2.20) is significantly higher than that in the overweight subgroup (OR: 1.16). Although MASLD is often associated with obesity, it is increasingly present in individuals with normal body mass index (BMI) and is sometimes referred to as “Thin MASLD”. It has been estimated that up to 20% of MASLD individuals have lean MASLD individuals (82). Recent studies have reported a higher risk of comorbidities, such as diabetes, hypertension, and cardiovascular disease, in lean MASLD patients compared with non-lean MASLD individuals (83–85). As mentioned previously, metabolic disorders, especially diabetes, may contribute to an increased risk of osteoporosis or osteoporotic fractures by affecting bone quality and bone microstructure (73–75). Furthermore, we have also mentioned above that the detrimental effect of MASLD on BMD may be offset by the positive effect of a higher BMI, but in normal-weight MASLD patients, the protective effect of a higher BMI on BMD is lost (62).

This meta-analysis has several limitations that need to be discussed. First, it is important to note that, as with any observational study, correlation does not equal causation. Second, the potential for residual confounding due to unmeasured variables related to BMD values, OP, or levels of BTMs cannot be entirely dismissed. Although the studies included in this analysis were adjusted for a variety of potential confounders, the possibility of residual confounding arising from factors (diet, physical activity, metabolic syndrome, vitamin D status, alcohol consumption, etc.) influencing bone health risk remains. Third, heterogeneity was evident in this meta-analysis, possibly due to differences in MASLD diagnosis, age, sex, weight, and region. Fourth, our meta-analysis included cohort and cross-sectional studies. Larger prospective studies are needed to assess the true impact of MASLD on bone metabolism or the risk of OP and osteoporotic fractures. Finally, in the section evaluating MASLD studies on BTMs, the included studies comprised only Asian populations (China, Korea, India), thus populations missing from other geographies, and studies that were not included limit the generalizability or extent to which the findings of this study can be applied to other situations, populations, or contexts, as there may be differences in the demographics and ethnic characteristics of the study populations, and further studies in multi-geographic populations are needed to evaluate the impact of MASLD on BTMs.

Despite these limitations, our meta-analytic study also has important strengths. First, the outcomes evaluated in this study were more comprehensive than previous meta-analyses, and we not only performed an analysis of the association of MASLD with BMD but also examined the effect of MASLD on the levels of BTMs and the risk of OP and osteoporotic fractures were further assessed. Additionally, on this basis, a number of subgroup analyses were also performed. Second, the present meta-analysis included a large number of participants, so sample size was adequate. Third, by adjusting the combined data for potential confounders, the confounders of the effects of MASLD on the risk of OP and osteoporotic fractures can be minimized, and therefore, conclusions based on this analysis may be valid, providing a basis for future research, and can improve the accuracy of risk assessment. Fourth, although the data showed high heterogeneity, we performed not only a sensitivity analysis but also a publication bias assessment, which further ensured the stability and reliability of the results.

In conclusion, our meta-analysis suggests that while MASLD is not associated with hip BMD and lumbar spine BMD values, it is positively associated with increased femoral BMD value, especially in the overweight and female populations. However, the prevalence of OP and osteoporotic fractures was significantly higher in MASLD than in non-MASLD individuals, regardless of sex, race or being overweight. Additionally, our study also found lower levels of BTMs in MASLD patients. This finding suggests that BMD may underestimate OP and osteoporotic fracture risk in MASLD patients. In addition, further research is needed to understand the underlying mechanisms and develop appropriate and effective interventions. Therefore, patients with MASLD should be regularly monitored for BMD values and levels of BTMs to prevent OP and osteoporotic fractures.
