# Supplementary material for: Association of metabolic dysfunction-associated steatotic liver disease with bone health in adults: a systematic review and meta-analysis of observational studies
Source: Front Endocrinol (Lausanne). 2026 Jan 12;16:1717852. doi: 10.3389/fendo.2025.1717852 (PMC12832396; doi:10.3389/fendo.2025.1717852)
Supplement: Supplementary file 1 [file Presentation1.pdf]

Non-alcoholic Fatty Liver Disease

- "Non alcoholic Fatty Liver Disease" or "Fatty Liver, Nonalcoholic" or "Fatty Livers, Nonalcoholic" or "Liver, Nonalcoholic Fatty" or "Livers, Nonalcoholic Fatty" or "Nonalcoholic Fatty Liver" or "Nonalcoholic Fatty Livers" or "NAFLD" or "Nonalcoholic Fatty Liver Disease" or "Nonalcoholic Steatohepatitis" or "Nonalcoholic Steatohepatitides" or "Steatohepatitides, Nonalcoholic" or "Steatohepatitis, Nonalcoholic"
- **Metabolism-related fatty liver disease (MAFLD)**
- **Metabolic dysfunction-associated steatotic liver disease (MASLD)**

- **Bone Density**
- "Bone Densities" or "Density, Bone" or "Bone Mineral Density" or "Bone Mineral Densities" or "Density, Bone Mineral" or "Bone Mineral Content" or "Bone Mineral Contents"

Osteoporosis

- "Osteoporoses" or "Osteoporosis, Age-Related" or "Osteoporosis, Age Related" or "Age-Related Osteoporosis" or "Age-Related Osteoporoses" or "Age Related Osteoporosis" or "Osteoporoses, Age-Related" or "Bone Loss, Age-Related" or "Age-Related Bone Loss" or "Age-Related Bone Losses" or "Bone Loss, Age Related" or "Bone Losses, Age-Related" or "Osteoporosis, Senile" or "Osteoporoses, Senile" or "Senile Osteoporoses" or "Senile Osteoporosis" or "Osteoporosis, Involutional" or "Osteoporosis, Post-Traumatic" or "Osteoporosis, Post Traumatic" or "Post-Traumatic Osteoporoses" or "Post-Traumatic Osteoporosis"

Bone Remodeling

"Remodeling, Bone" or "Bone Turnover" or "Bone Turnovers" or "Turnover, Bone" or "Turnovers, Bone" or "osteocalcin" or "Procollagen Type I n-terminal propeptide" or "Parathyroid hormone" or "C-terminal cross-linked telopeptide"
