# Supplementary material for: Association of metabolic dysfunction-associated steatotic liver disease with bone health in adults: a systematic review and meta-analysis of observational studies
Source: Front Endocrinol (Lausanne). 2026 Jan 12;16:1717852. doi: 10.3389/fendo.2025.1717852 (PMC12832396; doi:10.3389/fendo.2025.1717852)
Supplement: Supplementary file 5 [file Table4.docx]

Table S4 Publication bias of MASLD and BMD.

Table S5 Publication bias of MASLD and OP/ osteoporotic fractures.

1. CTX
2. OC


1. P1NP

1. PTH

Table S6 Publication bias of MASLD and BTMs.
