# Supplementary material for: Association of metabolic dysfunction-associated steatotic liver disease with bone health in adults: a systematic review and meta-analysis of observational studies
Source: Front Endocrinol (Lausanne). 2026 Jan 12;16:1717852. doi: 10.3389/fendo.2025.1717852 (PMC12832396; doi:10.3389/fendo.2025.1717852)
Supplement: Supplementary file 6 [file SupplementaryFile1.docx]

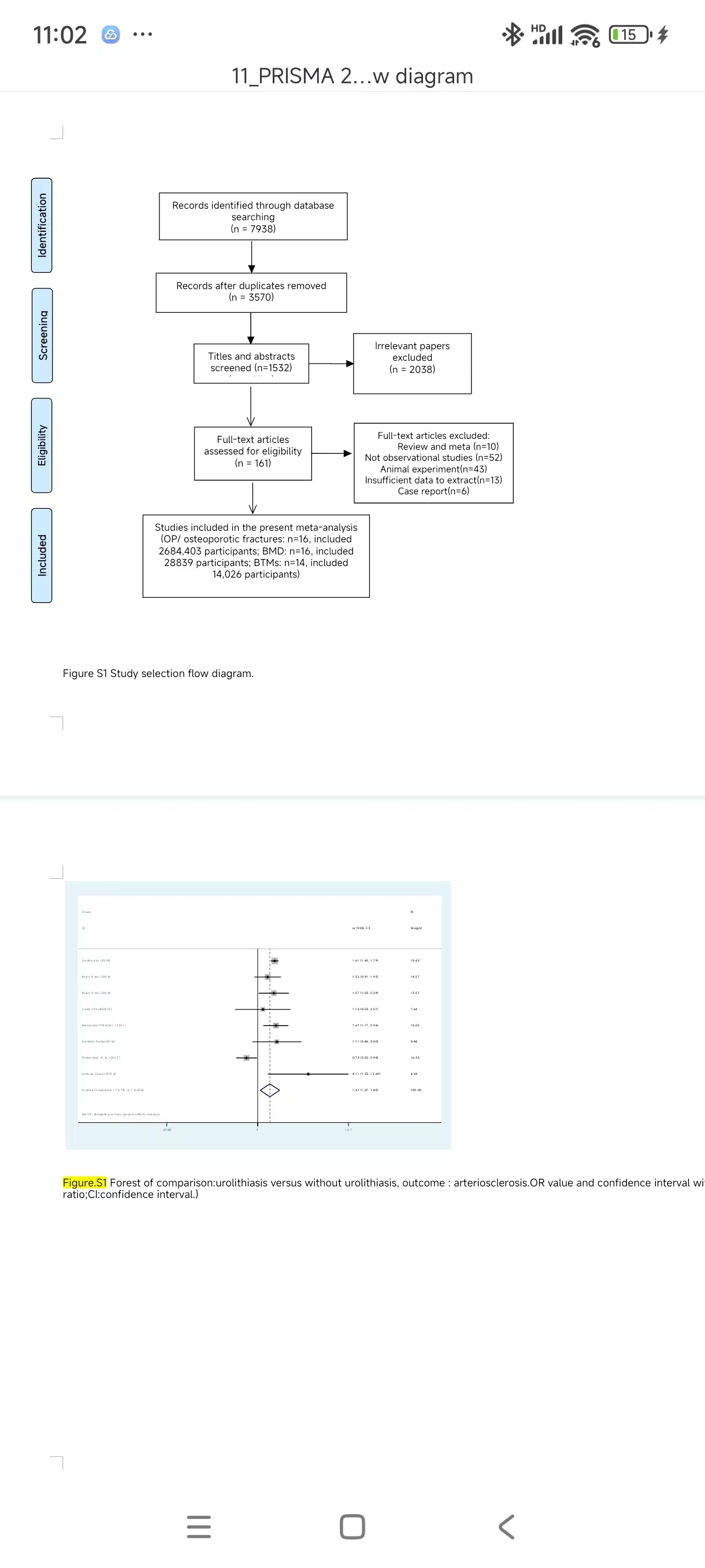


**Figure S1** Study selection flow diagram.

1. Forest plot of comparison of total BMD values at different gender between the MASLD and control groups.

1. Forest plot of comparison of femur BMD values at different gender between the MASLD and control groups.

1. Forest plot of comparison of hip BMD values at different gender between the MASLD and control groups.

1. Forest plot of comparison of lumbar spine BMD values at different gender between the MASLD and control groups.

**Figure S2** Forest plot of comparison of BMD values at different gender between the MASLD and control groups.

1. Forest plot of comparison of total BMD values at different region between the MASLD and control groups.

1. Forest plot of comparison of femur BMD values at different region between the MASLD and control groups.

1. Forest plot of comparison of hip BMD values at different region between the MASLD and control groups.

1. Forest plot of comparison of lumbar spine BMD values at different region between the MASLD and control groups.

**Figure S3** Forest plot of comparison of BMD values at different region between the MASLD and control groups.

（a）Forest plot of comparison of total BMD values at different weight between the MASLD and control groups.

(b) Forest plot of comparison of femur BMD values at different weight between the MASLD and control groups.

(c) Forest plot of comparison of lumbar spine BMD values at different weight between the MASLD and control groups.


（d）Forest plot of comparison of hip BMD values at different weight between the MASLD and control groups.

**Figure S4** Forest plot of comparison of BMD values at different weight between the MASLD and control groups.

**Figure S5** Forest plot of comparison of BMD values at different MASLD diagnostic modalities between the MASLD and control groups.

**Figure S6** Sensitivity analysis of MASLD and BMD

**Figure S7** Comparison of the OP/ osteoporotic fractures prevalence by gender.

**Figure S8** Comparison of the OP/ osteoporotic fractures prevalence by region.

**Figure S9** Comparison of the OP/ osteoporotic fractures prevalence by weight.

**Figure S10** Comparison of the OP/ osteoporotic fractures prevalence by diagnostic modalities of different MASLD.

**Figure S11** Sensitivity analysis of MASLD and OP/ osteoporotic fractures.

（a）CTX

(b) OC

（c）P1NP

(d) PTH

**Figure S12** Sensitivity analysis of MASLD and BTMs.

**Figure S13** Trim-and-fill evaluations between MASLD and OC.
